# SLC16A3 drives lung adenocarcinoma progression and gefitinib resistance through coordinated regulation of ferroptosis and lactate metabolism

**DOI:** 10.3389/fimmu.2025.1699540

**Published:** 2025-11-10

**Authors:** Wenhan Cai, Yiming Liu, Kai Zhao, Zirui Zhu, Jiamei Jin, Jiaxin Wen, Zhiqiang Xue

**Affiliations:** 1 Graduate School, Chinese PLA General Hospital, Beijing, China; 2 Department of Thoracic Surgery, First Medical Center, Chinese PLA General Hospital, Beijing, China; 3 Department of Thoracic Surgery, Hainan Hospital, Chinese PLA General Hospital, Sanya, China; 4 School of Medicine, Nankai University, Tianjin, China

**Keywords:** lung adenocarcinoma (LUAD), SLC16A3, ferroptosis, lactate metabolism, gefitinib resistance

## Abstract

**Background:**

Ferroptosis is an iron-dependent form of regulated cell death that plays a critical role in tumor suppression and therapeutic response. However, the metabolic mechanisms that drive ferroptosis resistance in lung adenocarcinoma (LUAD), particularly in the context of EGFR-TKI tolerance, remain unclear.

**Methods:**

We integrated transcriptomic and clinical data from TCGA LUAD cohort and performed survival and enrichment analyses. Functional assays, including proliferation, invasion, ferroptosis indicators, and *in vivo* xenograft models, were used to evaluate the role of SLC16A3. Lactate rescue, transcription factor prediction (JASPAR), mIHC, and luciferase reporter assays were used to dissect regulatory mechanisms. Pharmacological inhibition of SLC16A3 was used to assess its therapeutic potential.

**Results:**

SLC16A3 expression was elevated in LUAD and was correlated with poor prognosis. SLC16A3 knockdown suppressed tumor cell growth and enhanced ferroptosis, as indicated by increased lipid peroxidation, iron accumulation, and mitochondrial depolarization. Lactate supplementation partially reversed ferroptosis induction. Mechanistically, SLC16A3 was transcriptionally activated by HIF1A, and the HIF1A-SLC16A3 axis conferred ferroptosis resistance and gefitinib tolerance. SLC16A3 inhibition restored ferroptotic sensitivity and enhanced EGFR-TKI efficacy in xenograft models *in vivo*.

**Conclusion:**

Our findings reveal that the HIF1A-SLC16A3-lactate axis orchestrates ferroptosis suppression and therapeutic resistance in LUAD. Targeting SLC16A3 represents a promising metabolic strategy for overcoming EGFR-TKI resistance by reactivating ferroptosis.

## 1 Introduction

Lung adenocarcinoma (LUAD) is the most prevalent subtype of non-small cell lung cancer (NSCLC) and remains a leading cause of cancer-related mortality worldwide (1). Although targeted therapies such as epidermal growth factor receptor (EGFR) tyrosine kinase inhibitors (TKIs) have significantly improved clinical outcomes in patients with activating EGFR mutations, acquired resistance inevitably occurs, typically within a year of treatment initiation (2, 3). This resistance is driven not only by secondary EGFR mutations or bypass signaling but also by changes in cellular metabolism and stress adaptation (4, 5), underscoring the need to clarify resistance mechanisms and subsequent treatment options.

Lactate metabolism has emerged as a critical factor in tumor progression and therapeutic resistance. Lactate, once considered a mere metabolic byproduct, is now recognized as a signaling molecule that promotes angiogenesis, immune evasion, and cell survival (6, 7). Tumor cells rely on monocarboxylate transporters such as SLC16A3 (also known as MCT4) to export excess lactate and maintain the intracellular pH balance (8, 9). Elevated SLC16A3 expression is frequently observed in solid tumors and is correlated with poor prognosis (10, 11).

Ferroptosis, a form of iron-dependent non-apoptotic cell death driven by lipid peroxidation, has emerged as a critical regulator of tumor biology (12, 13). Unlike apoptosis, ferroptosis is closely linked to metabolic and redox homeostasis, and mounting evidence suggests that its evasion contributes to tumor growth, metastasis, and resistance to targeted therapies (13). In LUAD, ferroptosis is suppressed by multiple oncogenic pathways (14, 15), but the metabolic mechanisms that govern ferroptotic vulnerability remain incompletely defined. Notably, recent studies have proposed that suppression of ferroptosis may underlie resistance to EGFR-TKIs in non-small cell lung cancer, although the upstream regulators of this process are not fully understood (16). In parallel, lactate has been implicated as a metabolic modulator that buffers oxidative stress and protects cancer cells from ferroptosis (17, 18).

Based on these observations, we investigated the role of the lactate transporter SLC16A3 in linking lactate metabolism to ferroptosis resistance in LUAD. In this study, we identified SLC16A3 as a metabolic checkpoint that integrates lactate export and suppression of ferroptosis to promote LUAD progression and gefitinib tolerance. Using TCGA data, clinical samples, functional assays, and *in vivo* models, we showed that SLC16A3 overexpression was associated with poor prognosis and resistance phenotypes. Mechanistically, we demonstrated that SLC16A3-driven lactate export protects LUAD cells from ferroptosis and is transcriptionally regulated by hypoxia-inducible factor 1-alpha (HIF1A). These findings highlight the HIF1A-SLC16A3-lactate axis as a critical mediator of therapeutic escape and suggest SLC16A3 as a targetable metabolic vulnerability in LUAD.

## 2 Materials and methods

### 2.1 Data acquisition and bioinformatics analysis

Transcriptomic RNA-seq data and corresponding clinical annotations for patients with LUAD were downloaded from The Cancer Genome Atlas (TCGA) database (https://portal.gdc.cancer.gov/). Gene expression comparisons and Kaplan-Meier survival analyses were performed using R software (version 4.2.1). Differentially expressed genes (DEGs) were identified based on the thresholds of |log_2_FC|>1, FDR<0.05. KEGG pathway enrichment analysis was performed using Metascape (https://metascape.org), an integrated bioinformatics platform that combines multiple authoritative databases. Pathways with an adjusted P value (p.adj) < 0.05 and FDR < 0.25 were considered significantly enriched. Transcription factor-binding motif predictions for the SLC16A3 promoter were retrieved from the JASPAR database (https://jaspar.genereg.net/). Protein expression profiles of SLC16A3 in normal and LUAD tissues were assessed using the Human Protein Atlas (https://www.proteinatlas.org/). Clinicopathological characteristics of the TCGA-LUAD cohort used for correlation and survival analysis are summarized in **Table 1**.

**Table 1:** Baseline clinicopathological characteristics of LUAD patients stratified by SLC16A3 expression levels.

Clinicopathological feature	SLC16A3_low (n = 258)	SLC16A3_high (n = 258)	P_value
Survival status			0.006
Alive	180	149	
Dead	78	109	
Age Mean (SD)	65.7 (10.2)	64.9 (9.9)	0.393
Gender			0.331
Female	133	145	
Male	125	113	
Smoking history			
Non-smoking	40	35	0.54
Smoking	208	219	
T stage			0.017
T1	102	67	
T2	129	149	
T3	18	29	
T4	7	12	
TX	2	1	
N stage			0.008
N0	181	151	
N1	38	58	
N2	30	44	
N3	1	2	
NX	8	3	
M stage			0.405
M0	170	177	
M1	13	12	
MX	75	69	
TNM stage			0.019
I	157	122	
II	54	71	
III	33	52	
IV	14	13	

### 2.2 Clinical tissue samples

A total of 23 pairs of LUAD tumor tissues and matched adjacent normal lung tissues were collected from patients who underwent surgical resection at the Department of Thoracic Surgery, First Medical Center of the Chinese PLA General Hospital (Beijing, China). The histological and pathological diagnoses of all specimens were independently confirmed by at least two board-certified pathologists, according to the 2015 WHO classification of lung tumors. All patients provided written informed consent prior to enrollment, and the study was approved by the Institutional Review Board of the Chinese PLA General Hospital (Approval No. S2024-377-01). Immediately after resection, the samples were either snap-frozen in liquid nitrogen and stored at −80 °C or fixed in 10% neutral buffered formalin and paraffin-embedded. These tissues were used for western blotting, immunohistochemistry (IHC), and multiplex immunohistochemistry (mIHC). Clinicopathological characteristics of these 23 LUAD cases are summarized in **Table 2**.

**Table 2:** Baseline clinicopathological characteristics of the 23 LUAD patients included for gene expression correlation analysis.

Clinicopathological feature	n=23
Age (years, mean ± SD)	64.1 ± 8.3
Gender(Female/Male)	10/13
smoking history(Yes/No)	12/10
T stage	
T1	9
T2	9
T3	5
T4	1
N stage	
N0	12
N1	7
N2	4
M stage	
M0	23
TNM stage	
I	14
II	8
III	1

### 2.3 Cell culture and drug exposure

Human LUAD cell lines PC9, HCC827, and their gefitinib-resistant counterparts PC9GR and HCC827GR, as well as the normal bronchial epithelial cell line BEAS-2B, were obtained from the Meisen Chinese Tissue Culture Collection (Hangzhou, China). PC9 and PC9GR cells were cultured in RPMI-1640 medium (Gibco), whereas HCC827 and HCC827GR cells were maintained in Dulbecco’s Modified Eagle’s Medium (DMEM; Gibco). whereas HCC827 and HCC827GR cells were maintained in Dulbecco’s Modified Eagle’s Medium (DMEM; Gibco). BEAS-2B cells were cultured in DMEM (Gibco) under the same supplements. All cells were incubated at 37 °C in a humidified atmosphere containing 5% CO_2_ and were routinely confirmed to be free of mycoplasma contamination. Gefitinib, MSC-4381, ferrostatin-1 (Fer-1), Z-VAD-FMK, and chloroquine (CQ) were purchased from AbMole BioScience (Shanghai, China). All drugs were dissolved in dimethyl sulfoxide (DMSO) to prepare stock solutions and stored at -20°C. Working dilutions were freshly prepared in the culture medium prior to use, and control cells were treated with an equal concentration of DMSO.

### 2.4 RNA extraction and quantitative real-time PCR

Total RNA was extracted using the RNA-easy Isolation Kit (Vazyme, China) following the manufacturer’s instructions. Complementary DNA was synthesized using the HiScript^®^ III RT SuperMix for qPCR (Vazyme, China). Quantitative real-time PCR was performed using the ChamQ Universal SYBR qPCR Master Mix (Vazyme, China) on a QuantStudio 6 Flex Real-Time PCR System (Applied Biosystems, USA). GAPDH was used as an internal control, and relative mRNA expression was calculated using the 2^-ΔΔCt method. Primer sequences used are listed in **Supplementary Table S1**. Each condition was tested in triplicates (n = 3).

### 2.5 Gene silencing and overexpression

Stable knockdown or overexpression of SLC16A3 was achieved by lentiviral transduction using shRNA or full-length cDNA constructs. Lentiviral vectors carrying SLC16A3-targeting shRNAs (shSLC16A3#1/2/3) or control sequences (scramble) were purchased from Genechem (Shanghai, China) and cloned into the pLKO.1 vector. Packaging was performed in 293TN cells using psPAX2 and pMD2.G helper plasmids (Addgene) and the jetPRIME transfection reagent (Polyplus, France). The viral supernatants were harvested, filtered, and used to infect PC9 and PC9GR cells in the presence of polybrene (8 μg/mL). Stable clones were selected using puromycin (0.5 μg/mL) for 7 days. The efficiency of knockdown or overexpression was confirmed by qRT-PCR and western blotting.

For transient gene knockdown, small interfering RNAs (siRNAs) targeting SLC16A3 or candidate transcription factors (including HIF1A, KLF4, MXI1, RORA, ZNF460, and ZNF682) were synthesized by RiboBio (Guangzhou, China). Transfection was performed using Lipofectamine 3000 (Cat No. L3000015) in Opti-MEM (Invitrogen) according to the manufacturer’s protocol. Cells were harvested 48 h post-transfection for downstream assays. The sequences of all shRNAs and siRNAs are listed in **Supplementary Table S2**.

### 2.6 Western blotting

Cells or tissues were lysed using RIPA buffer (Beyotime, Shanghai, China) supplemented with protease and phosphatase inhibitor cocktails (Roche, Switzerland). The total protein concentration was determined using a BCA Protein Assay Kit (Thermo Fisher Scientific, USA). Equal amounts of protein (25 μg) were separated by SDS-PAGE and transferred to 0.45 μm PVDF membranes (Millipore, USA). The membranes were blocked with 5% non-fat milk for 2 h at room temperature and then incubated overnight at 4°C with primary antibodies against SLC16A3 (Proteintech), HIF1A (Abcam), SLC7A11 (Abcam), GPX4 (Abcam), FSP1 (Abcam), TFRC (Abcam), DHODH (Proteintech), and β-actin (Proteintech). After three washes with TBST, membranes were incubated with HRP-conjugated secondary antibodies (Cell Signaling Technology) at room temperature for 1 hour. Protein bands were visualized using ECL substrate (Thermo Fisher Scientific) and imaged using a chemiluminescence detection system. Band intensities were quantified using the ImageJ software and normalized to β-actin levels. All experiments were independently repeated in triplicate (n = 3). The antibody details are listed in **Supplementary Table S3**.

### 2.7 Cell proliferation and colony formation assays

For cell viability analysis, 5 × 10³ cells per well were seeded in 96-well plates and incubated overnight at 37°C with 5% CO_2_. The following day, the cells were treated with gradient concentrations of the indicated compounds for 24 h. Cell viability was assessed using the CCK-8 kit (Dojindo, Japan) according to the manufacturer’s instructions, and absorbance at 450 nm was measured to calculate relative viability. For colony formation assays, 500 cells per well were seeded in 6-well plates and cultured for 10–14 days. Colonies were fixed with 4% paraformaldehyde, stained with 0.1% crystal violet, and quantified using the ImageJ software. Each assay was performed in triplicates (n = 3).

### 2.8 Wound healing, migration and invasion assays

For the wound healing assay, PC9 and PC9GR cells were seeded in six-well plates and grown to confluence. A straight scratch was made using a sterile 200 μL pipette tip (0 h), and the wells were gently washed with PBS to remove detached cells. The cells were then cultured in serum-free medium and imaged at 0 and 24 h under a light microscope. Wound closure was quantified as the percentage of wound area reduction relative to 0 h using the ImageJ software.

Cell migration and invasion assays were performed using 24-well transwell chambers with 8 μm pore polycarbonate membrane inserts (Corning, USA). For the invasion assay, the upper chambers were pre-coated with Matrigel (BD Biosciences). Cells (5 × 10 unks for migration and 1 × 10 unks for invasion) were resuspended in 200 μL serum-free medium and seeded into the upper chambers. The lower chambers were filled with 500 μL of complete medium containing 15% FBS. After incubation for 24 h at 37°C and 5% CO_2_, non-migrated or non-invaded cells were removed from the upper surface using cotton swabs. Cells on the lower membrane surface were fixed with 4% paraformaldehyde and stained with 0.5% crystal violet (Solarbio, China). The migrated or invaded cells were imaged and counted in three randomly selected fields per insert.

### 2.9 Ferroptosis and cell death assays

Lipid peroxidation was assessed using BODIPY 581/591 C11 (Thermo Fisher Scientific), and mitochondrial membrane potential was measured using JC-1 dye (Sigma-Aldrich). Intracellular Fe^2+^ levels were detected using FerroOrange (MedChemExpress, MCE), according to the manufacturer’s protocol. To assess the role of different cell death pathways, cells were treated with ferrostatin-1 (2 µM), Z-VAD-FMK (10 µM), or chloroquine (10 µM) for 24 h, and cell viability was determined using the CCK-8 assay (Sigma-Aldrich). All experiments were performed in triplicate (n = 3).

### 2.10 Transmission electron microscopy

To assess the mitochondrial ultrastructure, cells were collected from 10 cm dishes after confirming interference efficiency and centrifuged at 4°C. The cell pellets were fixed overnight in 2.5% glutaraldehyde, followed by post-fixation with 1% osmium tetroxide and 2% uranyl acetate. After dehydration in graded ethanol and acetone, the samples were embedded in 812 epoxy resin (1:1 mixture with embedding agent for 2-4 h, followed by pure resin infiltration for 5-8 h at 37°C). Polymerization was performed at 60°C for 48 h. Ultrathin sections (60-80 nm) were prepared using an ultramicrotome, stained with uranyl acetate and lead citrate, and air dried. Ultrastructural images were acquired using a Hitachi HT7800 transmission electron microscope at 120 kV.

### 2.11 Lactate, glucose uptake, ATP, NADPH/NADP^+^, and GSH/GSSG quantification

Intracellular lactate levels were measured using a Lactate Assay Kit (Beyotime, China) following the manufacturer’s instructions. Intracellular glucose levels were detected using a Glucose Assay Kit based on the O-toluidine method (Beyotime). The cellular ATP content was quantified using an ATP Determination Kit (Beyotime). In addition, intracellular NADPH/NADP^+^ and GSH/GSSG ratios were quantified using commercial assay kits (ab65349; ab205811, Abcam, USA) according to the manufacturer’s instructions. Briefly, cells were lysed, deproteinized, and subjected to colorimetric detection at 450 nm. All values were normalized to total protein concentration. All measurements were conducted in triplicates (n = 3).

### 2.12 Animal experiments

All animal procedures were conducted in accordance with institutional guidelines and approved by the Institutional Animal Care and Use Committee of the Chinese PLA General Hospital. Female BALB/c nude mice (4–6 weeks old) were purchased from HFKBio (Beijing, China) and maintained under specific pathogen-free (SPF) conditions. PC9 cells (5 × 10^6^ in 100 μL PBS mixed 1:1 with Matrigel) were subcutaneously injected into the axillary region of each mouse. For genetic perturbation experiments, cells stably expressing shSLC16A3, OE-SLC16A3, shHIF1A, or the corresponding control vectors were used. For *in vivo* drug treatment, tumor-bearing mice were randomly divided into groups and treated with vehicle (DMSO), gefitinib (30 mg/kg), MSC-4381 (30 mg/kg), or a combination of both, via daily oral gavage. In the ferroptosis rescue group, ferrostatin-1 (5 mg/kg) was administered via intraperitoneal injection along with MSC-4381. Tumor volumes were measured every 4 days using calipers and calculated using the following formula: (length × width²)/2. On day 24 after injection, all mice were euthanized and tumors were collected for imaging, weighing, and further analyses. Each group consisted of four mice (n = 4).

### 2.13 Immunohistochemistry

Formalin-fixed, paraffin-embedded (FFPE) tumor tissues from xenograft models or clinical LUAD samples were sectioned at 4 μm thickness. The sections were deparaffinized, rehydrated, and subjected to antigen retrieval using citrate buffer (pH 6.0). Endogenous peroxidase activity was blocked using 3% hydrogen peroxide followed by nonspecific blocking with 5% BSA. After overnight incubation at 4°C with primary antibodies, including anti-SLC16A3 and anti-Ki-67, the sections were incubated with HRP-conjugated secondary antibodies. Immunoreactive signals were visualized using diaminobenzidine as chromogen. The slides were counterstained with hematoxylin and imaged using a Leica DM6 microscope. Two independent pathologists blinded to the group information scored immunostaining based on both the intensity and percentage of positive tumor cells. Staining intensity was graded as 0 (no staining), 1 (light yellow), 2 (yellow-brown), and 3 (deep brown), and the percentage of positive cells was scored as 0 (none), 1 (0-25%), 2 (26-50%), 3 (51-75%), and 4 (76-100%). The final immunoreactivity score was calculated as (Score1 × 1) + (Score2 × 2) + (Score3 × 3) with a maximum score of 300. The antibody details are provided in **Supplementary Table S4**, and all experiments were performed in triplicate (n = 3).

### 2.14 Multiplex immunohistochemistry

Formalin-fixed paraffin-embedded (FFPE) tumor tissues derived from xenograft models (shHIF1A and control groups) were sectioned at 4 μm thickness. Slides were deparaffinized in xylene (2 × 10 min), rehydrated through graded ethanol solutions (100%, 95%, and 70%; 5 min each), and subjected to antigen retrieval by microwave heating in citrate buffer (pH 6.0) for 15 min at 20% maximum power. After cooling and washing in Tris-buffered saline (TBS; pH 7.6; 3 × 5 min), sequential rounds of staining were performed using primary antibodies against HIF1A and SLC16A3. Each round included incubation with primary antibody (30 min, room temperature), HRP-conjugated secondary antibody, and signal development using the TSA Fluorescence Penta Staining Kit (RK05905, ReduX Biosciences, China). SLC16A3 and HIF1A signals were detected using the red and green fluorophores, respectively. Antigen retrieval was repeated between rounds to strip antibodies. Nuclei were counterstained with DAPI (Thermo Fisher), and the slides were coverslipped. Fluorescent images were captured using a Nikon A1R+ laser confocal microscope (Nikon, Japan), and quantitative analysis was performed using the inForm software (PerkinElmer). The antibody details are listed in **Supplementary Table S5**. All experiments were performed in duplicate using tumors from each group.

### 2.15 Dual-luciferase reporter assay

The wild-type (WT) and HIF1A-binding site mutant (Mut) promoter regions of the human SLC16A3 gene were cloned into the pGL3-Basic vector (Promega) upstream of the firefly luciferase gene. HEK293T cells were seeded into 24-well plates (4 × 10^4 cells/well) and co-transfected with 0.8 μg of pGL3 constructs, 0.02 μg of pRL-TK Renilla luciferase plasmid (internal control), and 0.8 μg of either pcDNA3.1-HIF1A or empty pcDNA3.1 vector using Lipofectamine™ 3000 (Thermo Fisher Scientific) according to the manufacturer’s protocol. After 48 h, cells were lysed and luciferase activity was measured using the Dual-Luciferase Reporter Assay System (Beyotime, RG088S) on a PerkinElmer Enspire plate reader. Firefly luciferase activity was normalized to Renilla luciferase activity, and relative promoter activity was calculated.

### 2.16 Statistical analysis

Statistical analyses were performed using GraphPad Prism 10 and R software (version 4.2.1). Data are presented as mean ± s.e.m. from at least three independent experiments. Differences between the two groups were analyzed using an unpaired Student’s t-test. One-way ANOVA was used for multiple group comparisons. Survival curves were analyzed using the Kaplan-Meier method and compared using the log-rank test. Univariate and multivariate Cox regression analyses were used to evaluate the prognostic factors. Significant differences are represented as *P < 0.05, **P < 0.01, ***P < 0.001, ****p < 0.0001, unless otherwise indicated.

## 3 Results

### 3.1 SLC16A3 is upregulated in lung adenocarcinoma and predicts poor prognosis

**Figure 1:**
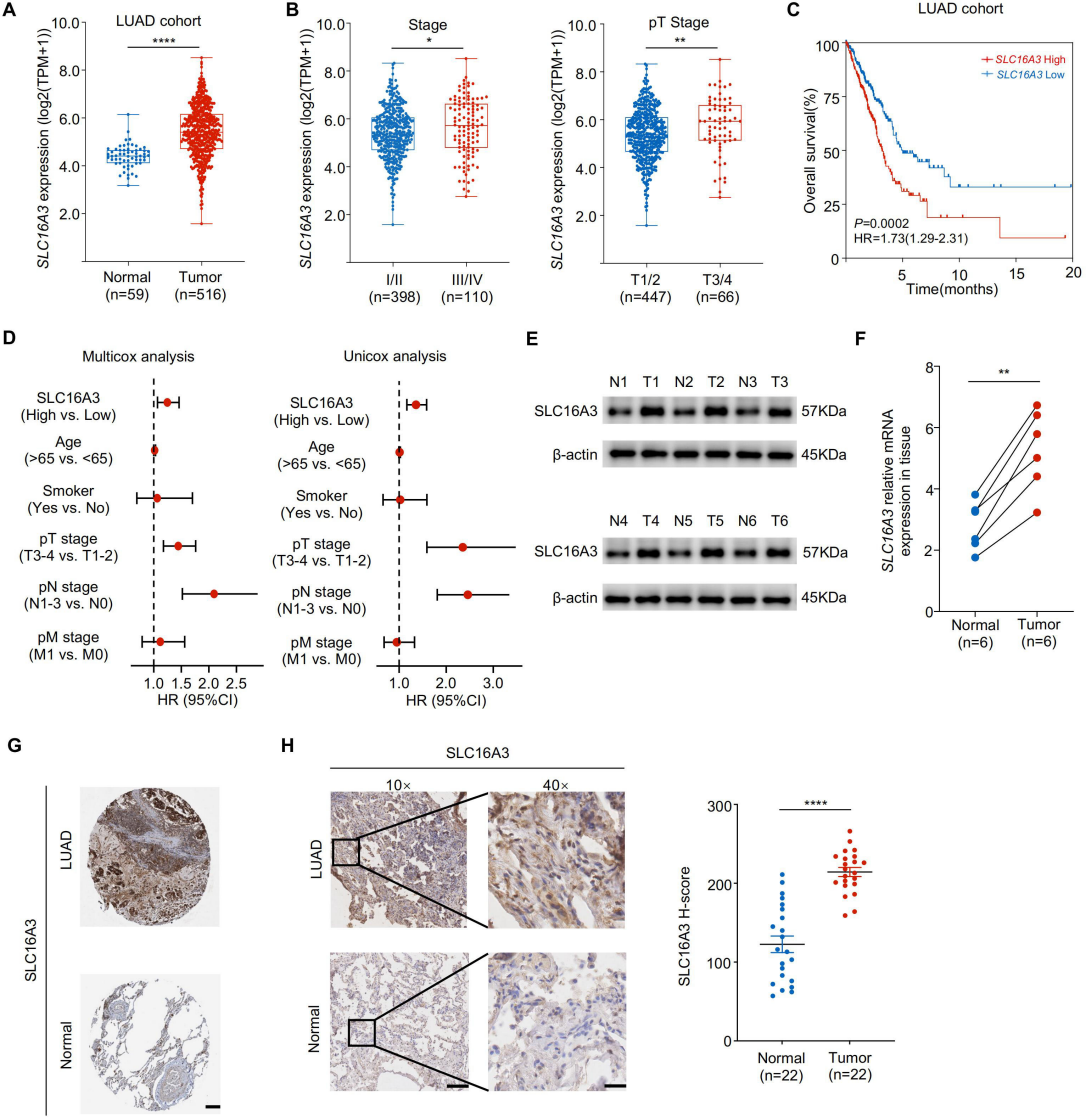
SLC16A3 is upregulated in lung adenocarcinoma and predicts poor prognosis. **(A)** SLC16A3 mRNA expression was significantly elevated in LUAD tumors (n = 516) compared with that in adjacent normal tissues (n = 59) from TCGA cohort. **(B)** Expression of SLC16A3 increased with advanced clinical stage (left) and pathological T stage (right). **(C)** Kaplan-Meier survival analysis of overall survival in patients with LUAD with high *vs.* low SLC16A3 expression. **(D)** Forest plots showing multivariate and univariate Cox regression analyses of clinical variables, including SLC16A3 expression, age, smoking status, and TNM staging. **(E)** Representative Western blot analysis of SLC16A3 protein levels in six paired LUAD tumors (T1-T6) and adjacent normal tissues (N1-N6). **(F)** Quantification of SLC16A3 protein levels from western blot data normalized to β-actin (n = 6). **(G)** Representative immunohistochemistry (IHC) staining images of LUAD and normal lung tissues obtained from the Human Protein Atlas (https://www.proteinatlas.org/). Scale bar, 500 µm. **(H)** IHC staining of clinical LUAD samples and paired adjacent normal tissues, confirming elevated SLC16A3 expression in tumors. Scale bars: 200 μm (10×), 50 μm (40×). Data are presented as mean ± s.e.m.; ns, not significant; *P < 0.05, **P < 0.01, ****P < 0.0001.

Analysis of transcriptomic data from TCGA LUAD cohort revealed that SLC16A3 expression was significantly upregulated in tumor tissues compared to that in adjacent normal tissues (**Figure 1A**). Stratification by clinical stage showed that SLC16A3 expression increased with both advanced clinical and pathological T stages (**Figure 1B**). Kaplan-Meier survival analysis demonstrated that high SLC16A3 expression was associated with worse overall survival in patients with LUAD (**Figure 1C**). Multivariate Cox regression analysis further confirmed that SLC16A3 is an independent prognostic factor for LUAD (**Figure 1D**).

Consistently, Western blot analysis of six paired LUAD and adjacent normal tissues confirmed elevated SLC16A3 protein expression in the tumors (**Figure 1E**). In parallel, qRT-PCR analysis validated the upregulated SLC16A3 mRNA levels in the tumor tissues (**Figure 1F**). Representative IHC images from the HPA database showed elevated SLC16A3 expression in LUAD tissues compared to that in normal lung tissues (**Figure 1G**), with IHC validation in our clinical LUAD cohort, further confirming enhanced SLC16A3 expression in tumor tissues compared to that in matched normal samples (**Figure 1H**).

### 3.2 SLC16A3 promotes LUAD cell proliferation, migration, invasion, and tumor growth

**Figure 2:**
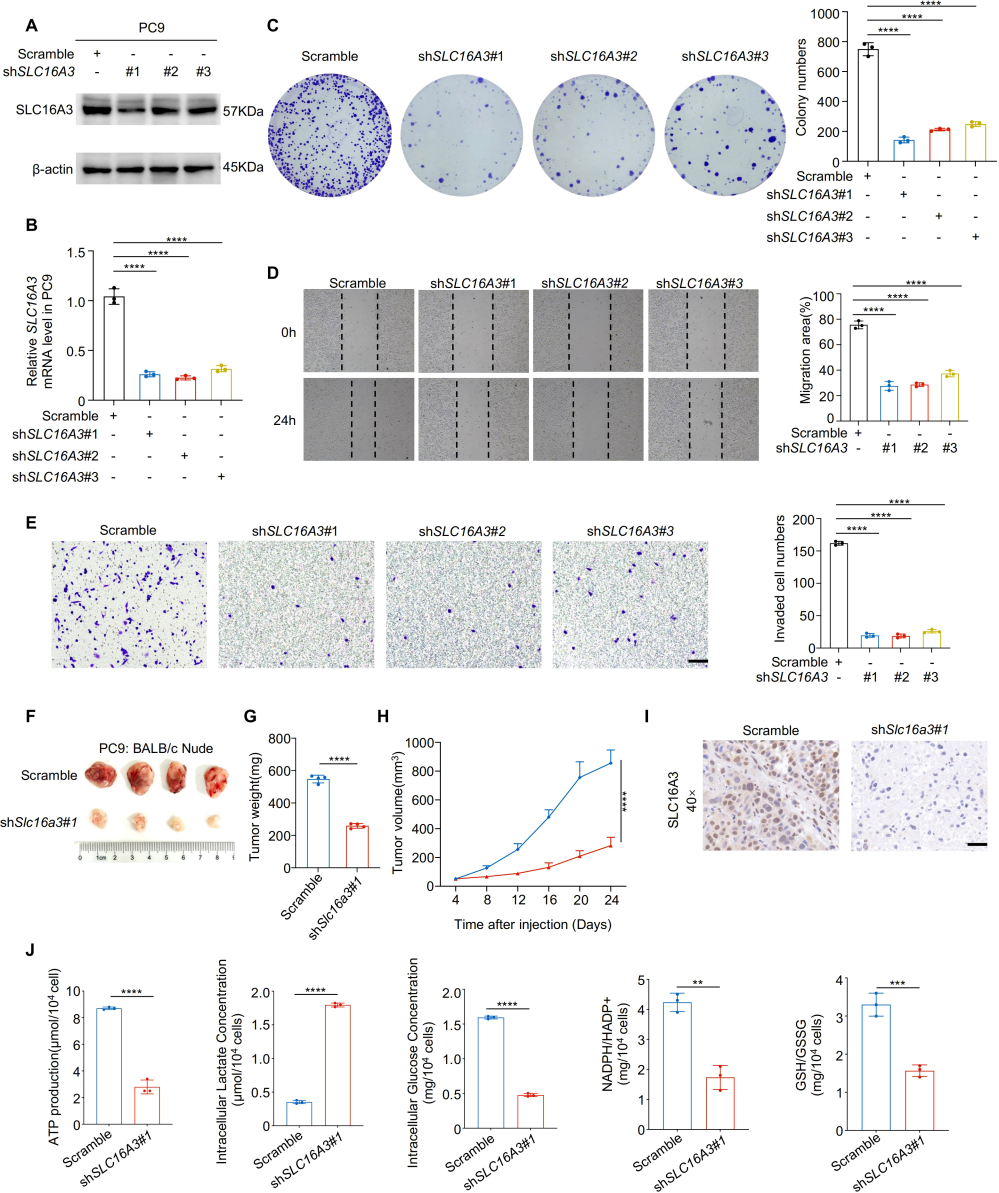
Knockdown of SLC16A3 suppresses lung adenocarcinoma cell proliferation, invasion, and tumorigenesis. **(A, B)** Western blot and qRT-PCR analysis confirming the knockdown efficiency of three independent SLC16A3 shRNAs in PC9 cells. **(C)** Colony formation assay showing reduced proliferative capacity upon SLC16A3 knockdown. **(D)** Wound healing assay evaluating cell migration after SLC16A3 silencing. **(E)** Transwell invasion assay showing significantly reduced invasiveness in shSLC16A3 cells. **(F–H)***In vivo* tumorigenesis assay using BALB/c nude mice injected subcutaneously with PC9 cells expressing scrambled shRNA or SLC16A3 shRNA (n = 4 per group). Scale bar, 100 µm. **(I)** IHC staining of SLC16A3 in the xenograft tissues. Scale bar, 50 µm. **(J)** Metabolic alterations upon SLC16A3 knockdown: intracellular ATP levels, intracellular and extracellular lactate production, intracellular glucose levels, and redox status indicators including NADPH/NADP^+^ and GSH/GSSG ratios in LUAD cells. Data are presented as mean ± s.e.m.; ns, not significant; **P < 0.01, ***P < 0.001, ****P < 0.0001.

To investigate the functional role of SLC16A3, we performed loss-of-function experiments in PC9 cells using three independent shRNAs. Efficient knockdown of SLC16A3 was confirmed using western blotting and qRT-PCR (**Figures 2A, B**). SLC16A3 silencing significantly impaired cell proliferation, as shown by colony formation assays (**Figure 2C**), and suppressed migration and invasion, as assessed by wound healing and Transwell assays (**Figures 2D, E**).

*In vivo* xenograft assays using BALB/c nude mice demonstrated that SLC16A3 knockdown markedly reduced tumor growth, as evidenced by the reduced tumor weight and volume over time (**Figures 2F–H**). IHC staining of xenograft tissues confirmed decreased SLC16A3 expression in shRNA-treated tumors (**Figure 2I**, **Supplementary Figure S1A**). Moreover, metabolic profiling revealed that SLC16A3 silencing markedly reduced intracellular ATP levels, lactate production, and glucose concentrations, together with lower NADPH/NADP^+^ and GSH/GSSG ratios (**Figure 2J**), reflecting disrupted redox homeostasis and diminished glycolytic capacity.

### 3.3 SLC16A3 overexpression enhances migration and invasion in LUAD cells

**Figure 3:**
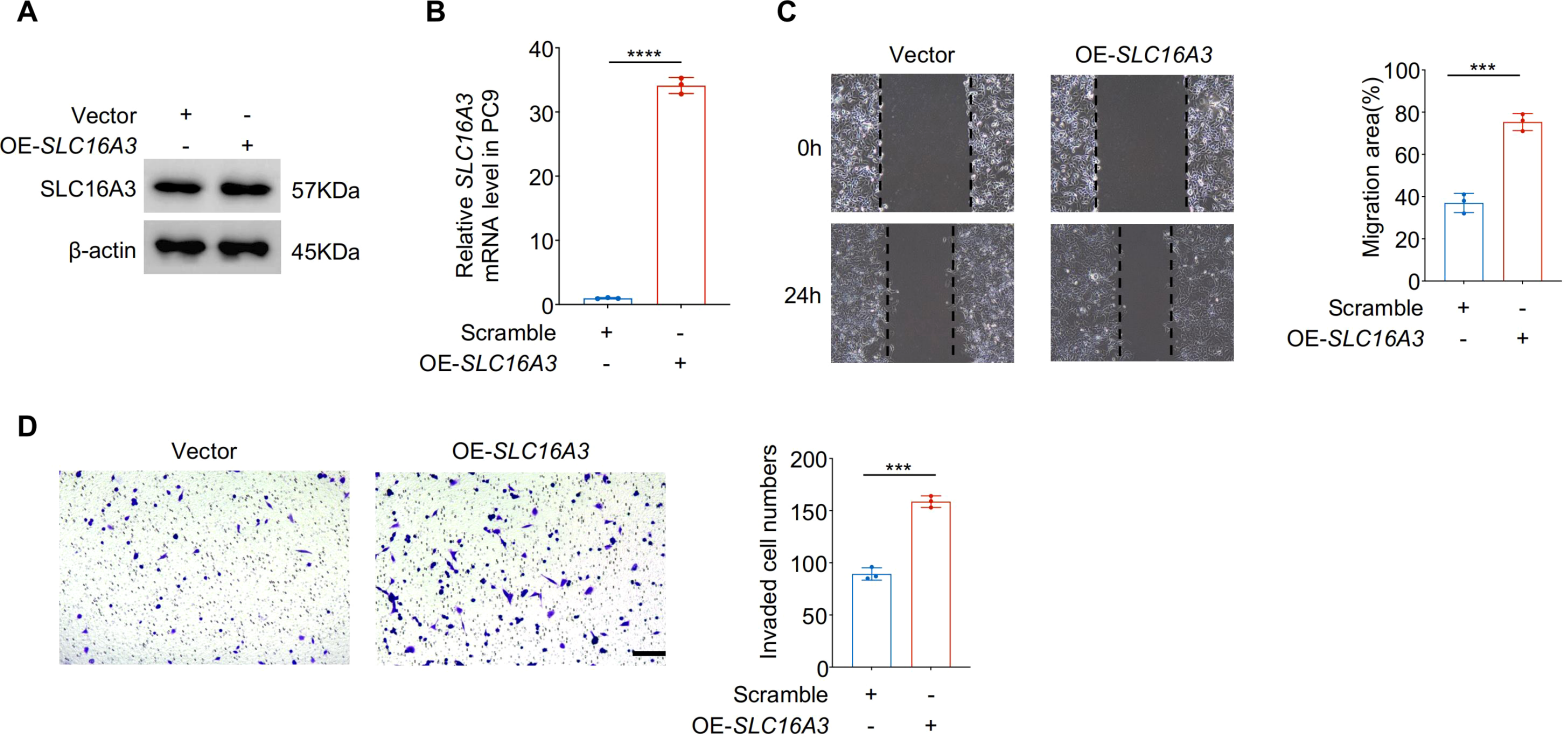
Overexpression of SLC16A3 enhances LUAD cell migration and invasion *in vitro*. **(A, B)** Validation of SLC16A3 overexpression in PC9 cells. Western blot **(A)** and qRT-PCR **(B)** analyses confirmed significantly elevated SLC16A3 protein and mRNA levels following transduction with the OE-SLC16A3 vector compared with the empty vector control. **(C)** Wound healing assay demonstrating increased migratory capacity in OE-SLC16A3 cells compared to the vector control. **(D)** Transwell invasion assay showing elevated invasive potential upon SLC16A3 overexpression. Scale bar, 100 µm. Data are presented as mean ± s.e.m.; ns, not significant; ***P < 0.001, ****P < 0.0001.

Gain-of-function studies in PC9 cells further validated the proinvasive function of SLC16A3. Western blotting and qRT-PCR confirmed successful overexpression of SLC16A3 (**Figures 3A, B**). Compared to the vector controls, SLC16A3-overexpressing cells exhibited significantly enhanced migration (**Figure 3C**) and invasion (**Figure 3D**), supporting the functional role of SLC16A3 in promoting malignant phenotypes.

### 3.4 SLC16A3 knockdown promotes ferroptosis via dysregulation of iron and redox homeostasis, and is partially modulated by lactate

**Figure 4:**
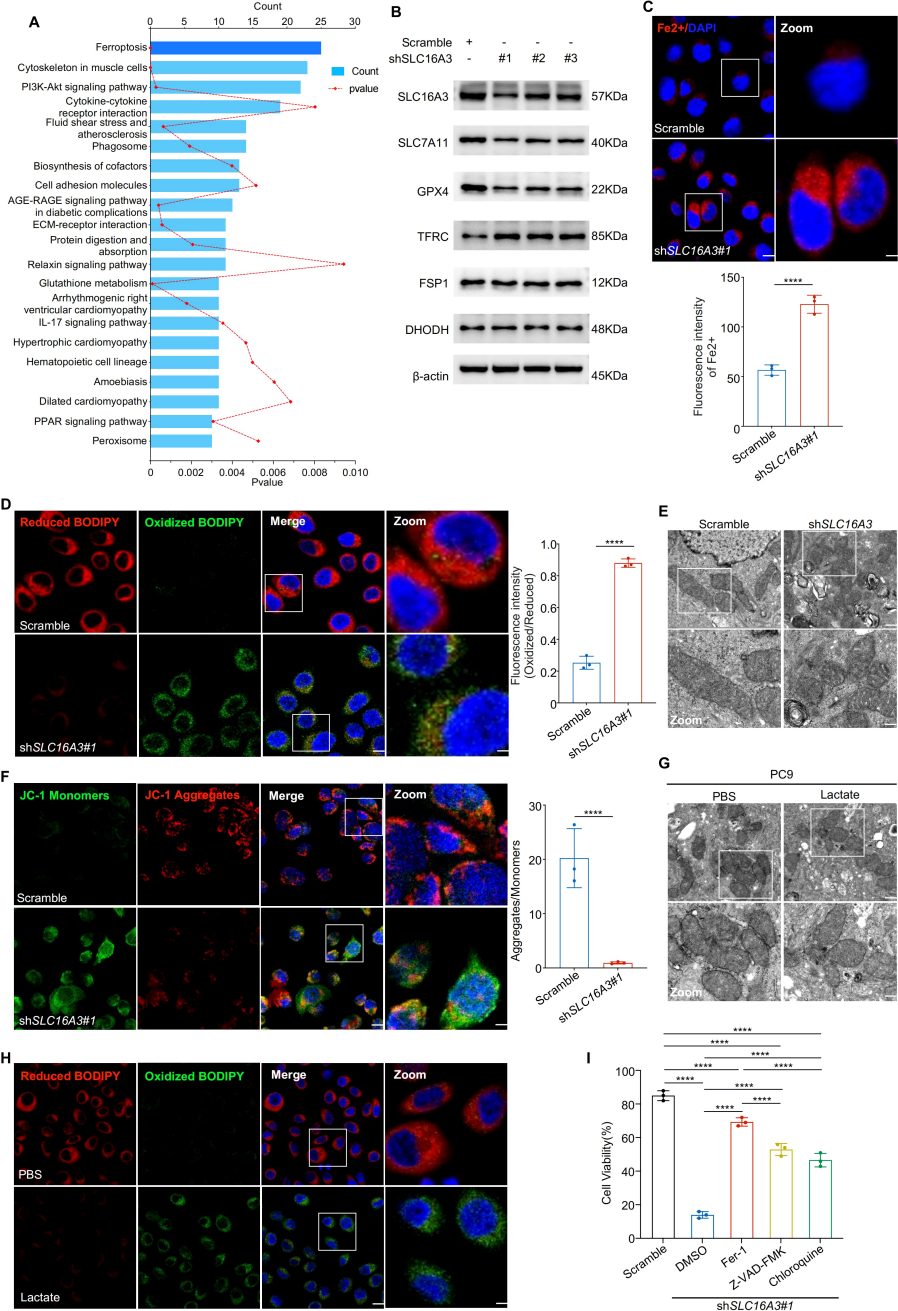
SLC16A3 knockdown promotes ferroptosis by disrupting iron and redox homeostasis, partially regulated by lactate. **(A)** KEGG enrichment analysis of differentially expressed genes upon SLC16A3 knockdown identified ferroptosis as the most enriched pathway. **(B)** Western blotting showing decreased expression of ferroptosis-protective proteins (SLC7A11, GPX4) and unchanged levels of FSP1, TFRC, and DHODH following SLC16A3 knockdown. **(C)** FerroOrange staining showing increased intracellular Fe^2+^ accumulation in shSLC16A3 cells; the right panel shows quantification of fluorescence intensity. Scale bars: 20 μm (left) and 5 μm (right). **(D)** C11-BODIPY staining showing increased lipid peroxidation in shSLC16A3 cells (green: oxidized, red: reduced); right panel quantifies the oxidized/reduced signal. Scale bars: 20 μm (left) and 5 μm (right). **(E)** Transmission electron microscopy reveals mitochondrial shrinkage, membrane density increase, and cristae loss in shSLC16A3 cells, consistent with ferroptotic morphology. Scale bars: 1 μm (top) and 500 nm (bottom). **(F)** JC-1 staining showing mitochondrial depolarization after SLC16A3 knockdown; bar graph shows the decreased aggregate/monomer ratio. Scale bars: 20 μm (left) and 5 μm (right). **(G)** TEM images of PC9 cells treated with PBS or lactate (10 mM), showing mitochondrial swelling, membrane disruption, and cristae reduction after lactate exposure. Scale bars: 1 μm (top) and 500 nm (bottom). **(H)** C11-BODIPY staining showing increased lipid peroxidation following lactate treatment in PC9 cells. Scale bars: 20 μm (left) and 5 μm (right). **(I)** Cell viability assay following treatment with ferrostatin-1 (Fer-1), Z-VAD-FMK, or chloroquine (CQ) in SLC16A3-silenced cells. Only Fer-1 showed substantial rescue. Data are presented as mean ± s.e.m.; ns, not significant; ****P < 0.0001.

To explore the mechanism by which SLC16A3 contributes to tumor progression, transcriptomic analysis was performed in PC9 cells stably expressing shSLC16A3 or the scramble control. KEGG enrichment analysis of differentially expressed genes revealed that ferroptosis was the most enriched pathway (**Figure 4A**). Western blot analysis confirmed that SLC16A3 knockdown reduced the expression of key ferroptosis defense proteins SLC7A11 and GPX4, whereas FSP1, TFRC, and DHODH levels remained largely unchanged (**Figure 4B**). Fluorescent staining with FerroOrange showed marked iron accumulation in shSLC16A3 cells (**Figure 4C**) and C11-BODIPY assays indicated enhanced lipid peroxidation (**Figure 4D**). Transmission electron microscopy revealed characteristic ferroptotic ultrastructural changes, including swollen mitochondria, disrupted outer membranes, and reduced or absent cristae (**Figure 4E**). JC-1 staining further confirmed mitochondrial depolarization (**Figure 4F**).

Given the known role of lactate in modulating the redox balance, we examined whether exogenous lactate could rescue ferroptosis induced by SLC16A3 silencing. Western blot analysis showed that lactate treatment reduced SLC7A11 and GPX4 expression (**Supplementary Figure S1B**). Lactate administration induced mitochondrial damage (**Figure 4G**) and increased lipid peroxidation (**Figure 4H**, **Supplementary Figure S1C**), indicating activation of ferroptosis. Finally, cell viability assays showed that apoptosis (Z-VAD-FMK), autophagy (CQ), and ferroptosis (ferrostatin-1) inhibitors reversed SLC16A3 knockdown-induced cell death to varying degrees, with ferrostatin-1 exhibiting the most pronounced protective effect (**Figure 4I**), indicating that ferroptosis was the predominant death mechanism downstream of SLC16A3 loss.

### 3.5 HIF1A transcriptionally regulates SLC16A3 and modulates ferroptosis sensitivity

**Figure 5:**
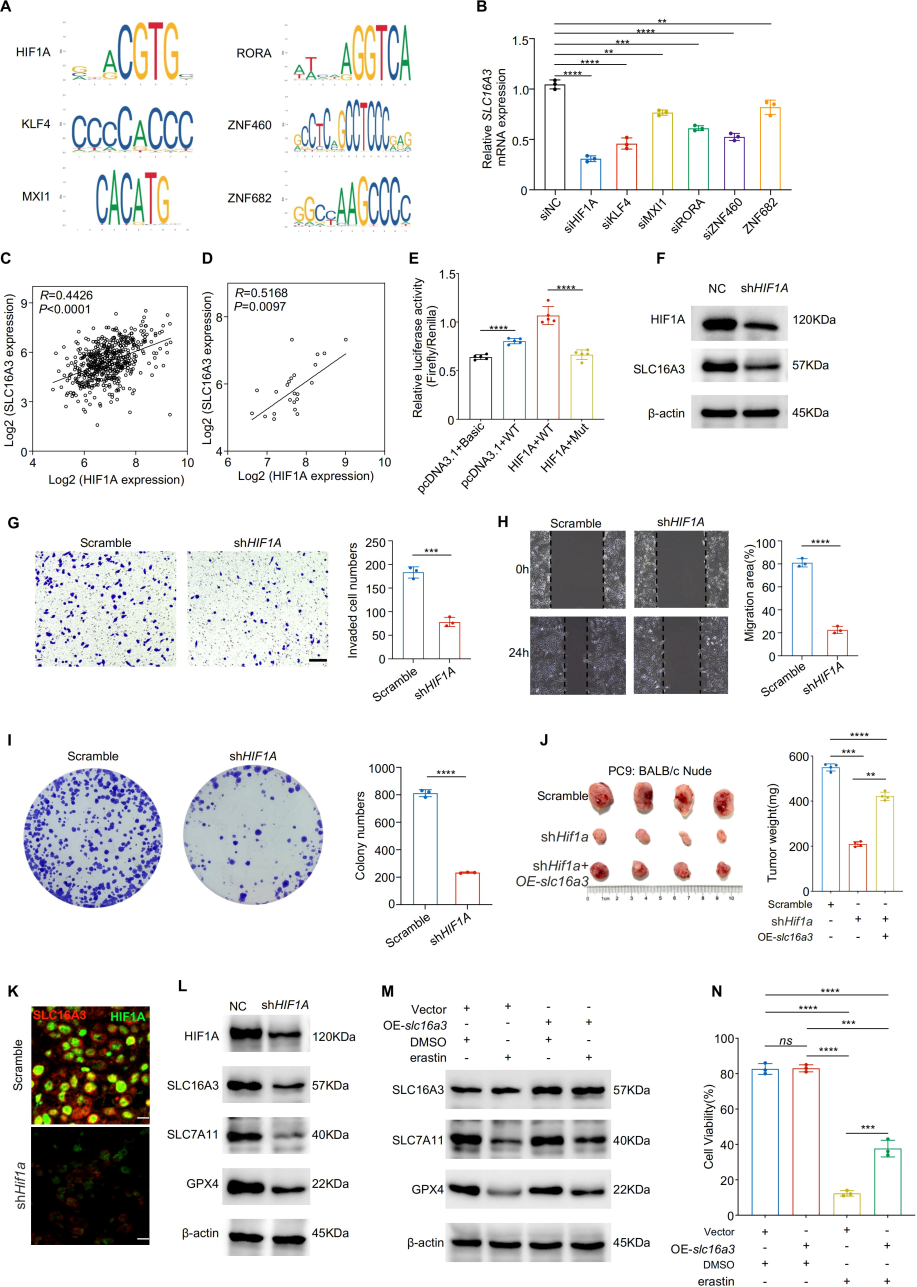
HIF1A transcriptionally activates SLC16A3 to promote malignant phenotypes and inhibit ferroptosis. **(A)** JASPAR analysis predicted multiple transcription factor binding motifs within the SLC16A3 promoter, including a binding site for HIF1A. **(B)** Knockdown screening identified that silencing HIF1A caused the greatest reduction in SLC16A3 mRNA expression among the tested transcription factors. C-D, HIF1A and SLC16A3 expression were positively correlated in both the TCGA-LUAD cohort **(C)** and in clinical LUAD samples **(D)**. **(E)** Dual-luciferase reporter assays were performed in four groups: (1) pcDNA3.1 + pGL3-Basic + TK (basal activity control); (2) pcDNA3.1 + pGL3-SLC16A3-prom (WT) + TK (wild-type promoter control); (3) HIF1A/pcDNA3.1 + pGL3-SLC16A3-prom (WT) + TK (HIF1A overexpression with WT promoter); and (4) HIF1A/pcDNA3.1 + pGL3-muthHRE-prom (Mut) + TK (HIF1A overexpression with mutated binding site). HIF1A overexpression significantly enhanced WT promoter activity, whereas mutation of the HIF1A-binding site markedly attenuated this effect. **(F)** Western blot analysis confirmed decreased SLC16A3 protein levels following HIF1A knockdown. G-I, Functional assays showed that HIF1A silencing suppressed invasion **(G)**, migration **(H)**, and colony formation **(I)** in LUAD cells. **(J)** In a subcutaneous xenograft model, shHIF1A reduced tumor growth, which was partially rescued by OE-slc16a3. **(K)** Multiplex immunofluorescence staining demonstrated colocalization of SLC16A3 and HIF1A in xenograft tumors, with SLC16A3 expression markedly decreased upon HIF1A silencing. **(L)** HIF1A knockdown reduced the ferroptosis defense proteins SLC7A11 and GPX4. **(M, N)**, Overexpression of slc16a3 restored SLC7A11 and GPX4 expression and maintained higher cell viability under erastin treatment. Data are presented as mean ± s.e.m.; ns, not significant; **P < 0.01, ***P < 0.001, ****P < 0.0001.

Transcription factor-binding motif analysis based on the JASPAR database predicted HIF1A as a potential regulator of SLC16A3 promoter activity (**Figure 5A**). The siRNA-mediated knockdown of HIF1A and several other predicted transcription factors reduced SLC16A3 mRNA expression, with HIF1A exhibiting the strongest inhibitory effect (**Figure 5B**). Expression correlation analyses of TCGA (**Figure 5C**) and clinical LUAD samples (**Figure 5D**) showed a strong positive correlation between HIF1A and SLC16A3.

Dual-luciferase reporter assays demonstrated that HIF1A overexpression significantly enhanced wild-type SLC16A3 promoter activity, whereas mutation of the HIF1A-binding site markedly attenuated this effect (**Figure 5E**). Western blotting confirmed that HIF1A knockdown reduced SLC16A3 protein expression (**Figure 5F**), consistent with decreased SLC16A3 mRNA levels observed by qPCR (**Supplementary Figure S1D**). mIHC further confirmed the colocalization of HIF1A and SLC16A3 in xenograft tumors, with SLC16A3 expression markedly reduced in shHIF1A tissues (**Figure 5K**), supporting the *in vivo* relevance of HIF1A-mediated transcriptional regulation.

Functionally, HIF1A knockdown suppressed cell invasion (**Figure 5G**), migration (**Figure 5H**), and colony formation (**Figure 5I**). SLC16A3 overexpression partially rescued the tumor-suppressive effect of HIF1A knockdown *in vivo* (**Figure 5J**). Mechanistically, HIF1A silencing downregulated the ferroptosis-protective proteins SLC7A11 and GPX4 (**Figure 5L**), which were restored by re-expression of SLC16A3. Moreover, SLC16A3 overexpression conferred resistance to erastin-induced ferroptosis (**Figure 5M**), as confirmed by cell viability assay (**Figure 5N**).

### 3.6 Targeting the HIF1A-SLC16A3 axis sensitizes gefitinib-resistant LUAD cells to ferroptosis

**Figure 6:**
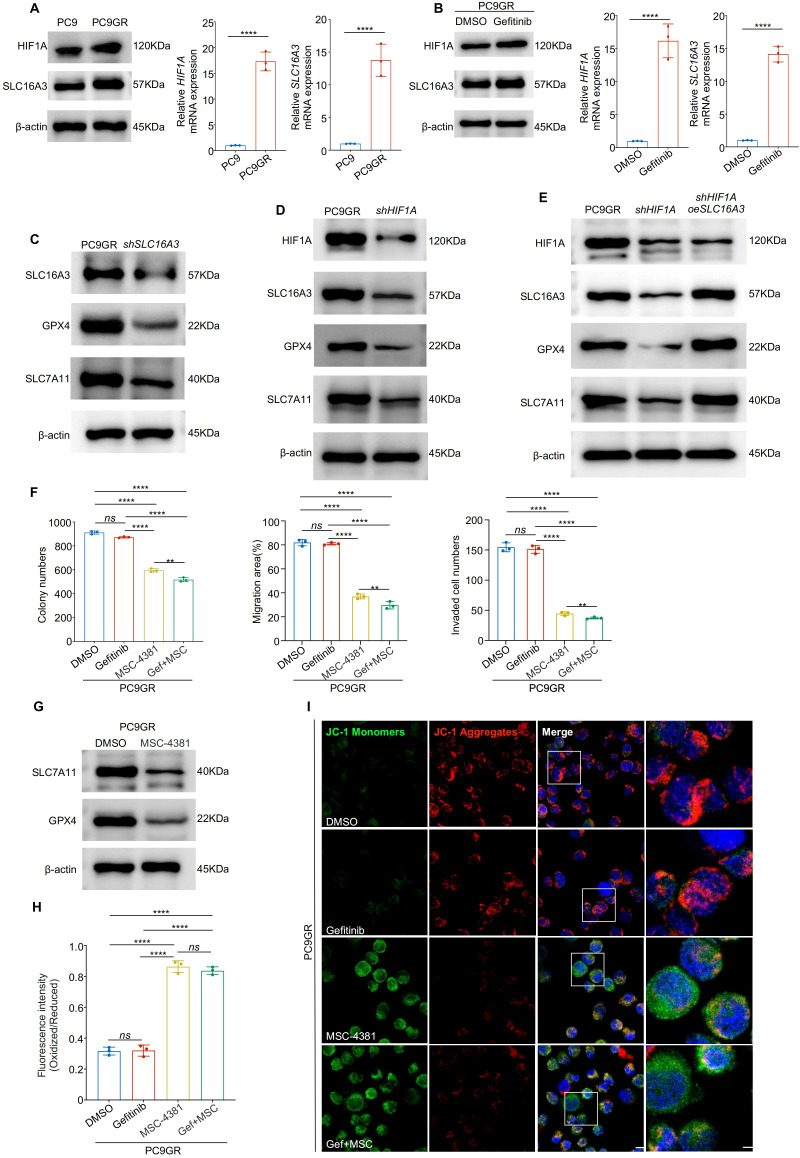
Targeting the HIF1A-SLC16A3 axis sensitizes gefitinib-resistant LUAD cells to ferroptosis **(A)** HIF1A and SLC16A3 expression was significantly upregulated in gefitinib-resistant PC9GR cells compared to parental PC9 cells, as shown by western blotting and qRT-PCR. **(B)** HIF1A and SLC16A3 are both upregulated following gefitinib treatment in PC9 cells. **(C)** Knockdown of SLC16A3 in PC9GR cells led to decreased expression of GPX4 and SLC7A11. **(D)** Knockdown of HIF1A similarly downregulates SLC16A3 and ferroptosis-related proteins. **(E)** Re-expression of SLC16A3 in shHIF1A cells restores GPX4 and SLC7A11 expression, confirming the regulatory role of the HIF1A-SLC16A3 axis. **(F)** Colony formation, wound healing, and transwell invasion assays showed that MSC-4381 sensitized PC9GR cells to gefitinib, suppressing cell proliferation and migration. **(G)** MSC-4381 treatment reduces SLC7A11 and GPX4 levels in PC9GR cells. **(H)** BODIPY-C11 staining indicates elevated lipid peroxidation upon gefitinib and MSC-4381 co-treatment. **(I)** JC-1 staining revealed mitochondrial depolarization in PC9GR cells treated with MSC-4381 and gefitinib, indicating ferroptosis-related damage. Scale bars: 20 μm (left) and 5 μm (right). Data are presented as mean ± s.e.m.; ns, not significant; **P < 0.01, ****P < 0.0001.

Next, we explored whether the HIF1A-SLC16A3 axis mediates resistance to gefitinib. Both HIF1A and SLC16A3 were significantly upregulated in gefitinib-resistant PC9GR cells compared with parental PC9 cells (**Figure 6A**) and were further induced by gefitinib treatment (**Figure 6B**). Silencing of either SLC16A3 (**Figure 6C**) or HIF1A (**Figure 6D**) downregulated GPX4 and SLC7A11 in PC9GR cells. The restoration of SLC16A3 rescued this effect (**Figure 6E**), confirming that HIF1A regulates ferroptosis via SLC16A3.

Functional assays showed that co-treatment with MSC-4381 (SLC16A3 inhibitor) and gefitinib significantly reduced colony formation, migration, and invasion of PC9GR cells compared to treatment with either agent alone (**Figure 6F**, **Supplementary Figure S1E-G**). Western blotting showed that MSC-4381 decreased GPX4 and SLC7A11 expression (**Figure 6G**) and BODIPY staining confirmed enhanced lipid peroxidation (**Figure 6H**, **Supplementary Figure S1H**). JC-1 assays indicated mitochondrial damage consistent with the induction of ferroptosis (**Figure 6I**, **Supplementary Figure S1I**). To further confirm the robustness of this mechanism, we validated the HIF1A-SLC16A3 axis in another EGFR-mutant LUAD model (HCC827/HCC827GR). Both HIF1A and SLC16A3 were markedly upregulated in resistant cells and further induced by gefitinib treatment (**Supplementary FigureS S2A, B**). Silencing of HIF1A significantly decreased SLC16A3 expression, supporting the transcriptional regulation of this axis (**Supplementary Figure S2C**). Functionally, co-treatment with gefitinib and MSC-4381 increased intracellular Fe^2+^ accumulation (**Supplementary Figure S2D**) and lipid ROS levels (**Supplementary Figure S2E**), as evidenced by FerroOrange and BODIPY staining, indicating ferroptosis activation in HCC827GR cells.

### 3.7 Pharmacological inhibition of SLC16A3 enhances gefitinib efficacy *in vivo* through ferroptosis induction

**Figure 7:**
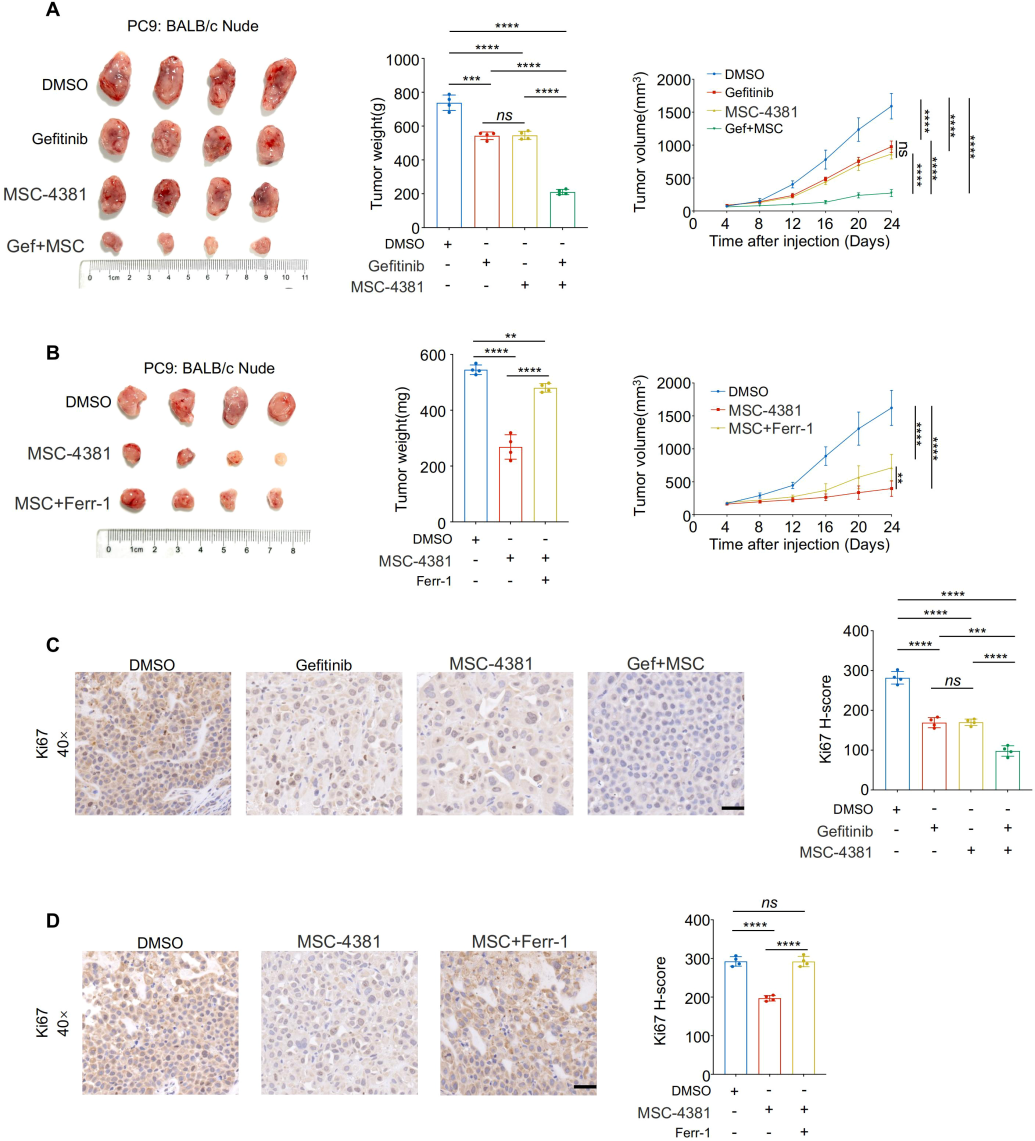
Pharmacological inhibition of SLC16A3 enhances gefitinib efficacy *in vivo* through ferroptosis induction. **(A)** An *in vivo* xenograft tumor model using PC9 cells in BALB/c nude mice. Mice were treated with DMSO, gefitinib, MSC-4381 (SLC16A3 inhibitor), or their combination (G + M). Representative tumor images, final tumor weights, and tumor volume curves are shown (n = 4 per group). Co-treatment with gefitinib and MSC-4381 significantly suppressed tumor growth compared with monotherapy. **(B)** MSC-4381-mediated tumor inhibition was reversed by co-administration of the ferroptosis inhibitor ferrostatin-1, confirming ferroptosis dependence. **(C)** Immunohistochemical staining of Ki-67 in tumor tissues from the treatment groups in **(A)** showing a reduced proliferative index upon combination therapy. Scale bars, 50 μm. **(D)** Ki-67 IHC in tissues from the ferrostatin-1 rescue experiment in **(B)**, showing that ferroptosis inhibition restores proliferative capacity suppressed by MSC-4381. Scale bars, 50 μm. Data are presented as mean ± s.e.m.; ns, not significant; **P < 0.01, ***P < 0.001, ****P < 0.0001.

To validate the therapeutic potential of SLC16A3 inhibition *in vivo*, PC9 xenograft-bearing mice were treated with MSC-4381, gefitinib, or both. Co-treatment significantly suppressed tumor growth compared with treatment with either agent alone (**Figure 7A**). Ferrostatin-1 partially attenuated the antitumor effect of MSC-4381, confirming that ferroptosis is a key mechanism (**Figure 7B**). IHC staining showed that co-treatment reduced Ki-67 expression, which is indicative of impaired proliferation (**Figure 7C**), whereas ferroptosis inhibition restored Ki-67 positivity (**Figure 7D**).

## 4 Discussion

Ferroptosis, a regulated form of non-apoptotic cell death driven by iron-dependent lipid peroxidation, has emerged as a critical vulnerability in cancer therapy (19, 20). In LUAD, ferroptosis is often suppressed to promote tumor survival and therapy resistance (21, 22). Although key regulators such as GPX4, SLC7A11, and FSP1 have been widely studied (23–25), the metabolic cues that govern ferroptotic evasion remain incompletely defined. Our study identified SLC16A3, a lactate exporter, as the central metabolic node that suppresses ferroptosis and contributes to EGFR-TKI resistance in LUAD.

SLC16A3 (also known as MCT4) is a hypoxia-inducible transporter that facilitates lactate efflux during aerobic glycolysis (26). Its overexpression has been correlated with tumor aggressiveness, metastasis, and poor prognosis across multiple cancers, including breast, prostate, and lung cancer (27, 28). In line with previous reports, we showed that SLC16A3 was upregulated in LUAD tumors and correlated with advanced clinical stages and poor outcomes. While most studies have focused on its role in pH regulation and metabolic adaptation, we have now demonstrated its capacity to regulate ferroptosis by maintaining redox balance through lactate export. To further support this mechanism, we additionally quantified key intracellular redox indicators. Specifically, both the NADPH/NADP^+^ and GSH/GSSG ratios were significantly reduced following SLC16A3 knockdown, confirming that inhibition of SLC16A3 disrupts redox homeostasis. These findings provide direct metabolic evidence that SLC16A3-mediated lactate export contributes to the maintenance of cellular redox balance, thereby sustaining ferroptosis resistance. Mechanistically, we further confirmed that HIF1A directly binds to and activates the SLC16A3 promoter, as evidenced by dual-luciferase reporter assays and qPCR validation following site-directed mutagenesis of the HIF1A-binding site. This direct transcriptional regulation provides a mechanistic link between hypoxia signaling and SLC16A3-mediated ferroptosis suppression in LUAD. Importantly, our study reveals that this regulatory axis operates under normoxic conditions, without the need for exogenous hypoxia stimulation. While previous studies have associated lactate accumulation or hypoxic adaptation with ferroptosis resistance, they have not established a direct transcriptional link between HIF1A and SLC16A3 in LUAD. Here, we provide the first evidence that HIF1A directly activates SLC16A3 transcription in normoxia, leading to enhanced lactate export, maintenance of redox homeostasis, and ferroptosis evasion. Moreover, by extending our validation to an independent EGFR-mutant LUAD model (HCC827/HCC827GR), we further underscored the robustness and generalizability of this mechanism. Consistent regulatory patterns—namely, HIF1A-dependent upregulation of SLC16A3 and ferroptosis sensitization upon SLC16A3 inhibition—suggest that the HIF1A-SLC16A3 axis represents a conserved adaptive program across distinct genetic backgrounds of EGFR-driven LUAD. This cross-model consistency not only reinforces the mechanistic link between lactate metabolism and ferroptotic resistance but also implies that targeting SLC16A3 could provide therapeutic benefit beyond a single cellular context. Furthermore, our results demonstrate that SLC16A3 contributes to EGFR-TKI resistance through metabolic dysregulation, extending the functional significance of lactate metabolism from energy adaptation to therapeutic response regulation.

Recent evidence has linked lactate accumulation to ferroptosis resistance through multiple mechanisms, including NADPH generation, glutathione maintenance, and lipid remodeling (29–31). Lactate can serve as an alternative carbon source for NADH/NADPH production under stress conditions, thereby counteracting lipid ROS accumulation (32). Our findings support this metabolic buffering model, showing that SLC16A3 inhibition enhances lipid peroxidation, depletes ferroptosis-protective proteins (GPX4 and SLC7A11), and disrupts mitochondrial integrity. Consistent with these observations, SLC16A3 knockdown markedly reduced the NADPH/NADP^+^ and GSH/GSSG ratios, indicating that impaired lactate efflux compromises intracellular redox homeostasis. NADPH acts as a critical reducing equivalent for GSH regeneration, while GSH is indispensable not only for GPX4 enzymatic activity but also for maintaining SLC7A11 stability under oxidative stress. Thus, SLC16A3-mediated lactate export safeguards ferroptosis resistance by sustaining NADPH supply and GSH recycling, thereby stabilizing the GPX4/SLC7A11 antioxidant defense system. In line with this mechanism, previous studies have shown that blockade of lactate efflux causes intracellular acidification and ROS accumulation, which aggravate oxidative stress and activate ferroptotic signaling (33) Beyond redox regulation, emerging evidence suggests that lactate metabolism may also influence ferroptosis via epigenetic mechanisms such as histone lactylation, which modulates the transcriptional activity of ferroptosis-related genes (34). Collectively, these findings reveal that SLC16A3 protects LUAD cells from ferroptosis through both metabolic and epigenetic modulation of the GPX4/SLC7A11 axis. Consistent with this conclusion, ultrastructural features of ferroptosis, including condensed mitochondria and cristae loss, were observed upon SLC16A3 knockdown, reinforcing its role in ferroptotic regulation. Among several death pathway inhibitors, only ferrostatin-1 markedly rescued cell viability, excluding the contributions from apoptosis, autophagy, or necroptosis. Together, these results highlight SLC16A3-mediated lactate export as a previously underappreciated metabolic brake on ferroptosis in LUAD.

Pharmacological inhibition of SLC16A3 using MSC-4381 phenocopied genetic knockdown impaired redox homeostasis and sensitized LUAD cells to ferroptosis. Moreover, MSC-4381 synergized with gefitinib *in vitro* and *in vivo*, and this effect was abrogated by ferrostatin-1, confirming ferroptosis as the mechanistic basis of combination therapy. These findings echo recent studies showing that ferroptosis induction enhances the efficacy of EGFR inhibitors and overcomes acquired resistance (35). Notably, MSC-4381 is a structurally optimized and pharmacologically validated selective inhibitor of SLC16A3, exhibiting over 50-fold selectivity relative to MCT1 and MCT2 (36). Its target specificity and *in vivo* efficacy have been further supported by multiple independent studies, in which MSC-4381 effectively blocked SLC16A3-mediated lactate oxidation and suppressed tumor progression without detectable off-target effects (37, 38). Consistent with these reports, our data showed that MSC-4381 treatment phenocopied the effects of SLC16A3 knockdown, including reduced lactate export, impaired redox balance, and ferroptosis activation, thereby confirming that the observed antitumor activity is primarily dependent on SLC16A3 inhibition.

Our results support a growing paradigm in which targeting metabolic regulators restores ferroptosis sensitivity in therapy-resistant tumors. In particular, the dual role of SLC16A3 in lactate clearance and suppression of ferroptosis is a promising target in LUAD. However, further studies are required to confirm this hypothesis. Although MSC-4381 exhibited robust antitumor efficacy in subcutaneous xenografts, future studies employing orthotopic or patient-derived xenograft (PDX) models may better substantiate its therapeutic relevance within the native lung microenvironment. Moreover, whether the HIF1A-SLC16A3-ferroptosis axis extends to other oncogenic backgrounds, such as KRAS- or ALK-driven LUAD, remains an interesting direction for future investigation.

From a translational perspective, our findings suggest that targeting SLC16A3 may represent a potential adjunct strategy to enhance the efficacy of EGFR-TKIs in LUAD. Given the metabolic role of SLC16A3 in maintaining redox homeostasis, its inhibition could sensitize tumor cells to ferroptosis under EGFR-TKI treatment. Although MSC-4381 has demonstrated selective inhibition of SLC16A3 with favorable preclinical tolerability, comprehensive pharmacokinetic and safety evaluations are still required before clinical application. Future studies integrating SLC16A3 inhibitors with established EGFR-TKI regimens, as well as optimizing dosing schedules and assessing potential off-target effects, will be critical for validating its therapeutic relevance.

In conclusion, this study revealed a metabolic axis linking HIF1A-induced SLC16A3 expression to ferroptosis suppression and EGFR-TKI resistance via lactate export. By showing that SLC16A3 inhibition restores ferroptotic sensitivity and synergizes with EGFR-targeted therapy, we highlight its potential as a metabolic target for overcoming therapeutic resistance in LUAD.

## 5 Conclusion

This study reveals the HIF1A-SLC16A3 axis is a key mediator of ferroptosis resistance and EGFR-TKI tolerance in lung adenocarcinoma. By linking lactate metabolism to ferroptosis suppression, we uncovered a metabolic mechanism for therapeutic escape. Targeting SLC16A3 restores ferroptosis sensitivity and enhances gefitinib efficacy in resistant models, suggesting that it is clinically actionable metabolic vulnerability. These findings provide a rationale for the development of ferroptosis-based combination strategies for LUAD.

## Data Availability

The datasets presented in this study can be found in online repositories. The names of the repository/repositories and accession number(s) can be found in the article/**Supplementary Material**.

## Supplementary material

Supplementary Figure 1 Supporting data for ferroptosis regulation by SLC16A3 and therapeutic synergy of MSC-4381 with gefitinib. **(A)** Quantification of SLC16A3 immunohistochemical staining (H-score) in xenograft tumors from the scramble and shSLC16A3 groups (corresponding to **Figure 2I**). **(B)** Western blot analysis of ferroptosis-related proteins SLC7A11 and GPX4 in PC9 cells treated with exogenous lactate (10 mM) (corresponding to **Figure 4H**). **(C)** Quantification of lipid peroxidation (oxidized/reduced BODIPY C11 ratio) in lactate-treated PC9 cells (corresponding to **Figure 4H**). **(D)** qPCR analysis of SLC16A3 mRNA expression following HIF1A knockdown in PC9 cells (corresponding to **Figure 5F**). **(E–G)** Representative images of colony formation (E; corresponding to **Figure 6F**), wound healing (F; corresponding to **Figure 6G**), and Transwell invasion (G; corresponding to **Figure 6H**) assays in DMSO, gefitinib, MSC-4381, or gefitinib + MSC-4381 treatments. Scale bars, 100 μm. **(H)** Representative images of C11-BODIPY staining showing lipid ROS levels after the indicated treatments (corresponding to **Figure 6H**). Scale bars: 20 μm (left) and 5 μm (right). **(I)** Quantification of JC-1 red/green fluorescence ratio, indicating mitochondrial membrane potential changes (corresponding to **Figure 6I**). Data are presented as mean ± s.e.m.; ns, not significant; **P < 0.01, ***P < 0.001, ****P < 0.0001.

Supplementary Figure 2 Validation of the HIF1A-SLC16A3 axis and ferroptosis activation in an additional EGFR-mutant LUAD model (HCC827/HCC827GR). **(A)** qPCR analysis showing elevated HIF1A and SLC16A3 mRNA expression in HCC827GR compared with HCC827 cells (n = 3). **(B)** HIF1A and SLC16A3 mRNA levels were upregulated following gefitinib treatment in HCC827GR cells (n = 3). **(C)** Knockdown of HIF1A significantly decreased SLC16A3 expression in both HCC827 and HCC827GR cells (n = 3). **(D)** FerroOrange staining showing increased Fe2+ accumulation in HCC827GR cells upon gefitinib and MSC-4381 co-treatment (scale bar = 10 μm). Quantification of fluorescence intensity is shown below (n = 3). **(E)** C11-BODIPY 581/591 staining showing enhanced lipid peroxidation (oxidized/reduced ratio) after gefitinib and MSC-4381 co-treatment (scale bar = 10 μm). Quantification of fluorescence ratio is shown below (n = 3). Data are presented as mean ± SEM; ns, not significant; **P < 0.01, ***P < 0.001, ****P < 0.0001.
