# Supplementary figures and images for: SLC16A3 drives lung adenocarcinoma progression and gefitinib resistance through coordinated regulation of ferroptosis and lactate metabolism
Source: Front Immunol. 2025 Nov 10;16:1699540. doi: 10.3389/fimmu.2025.1699540 (PMC12640836; doi:10.3389/fimmu.2025.1699540)

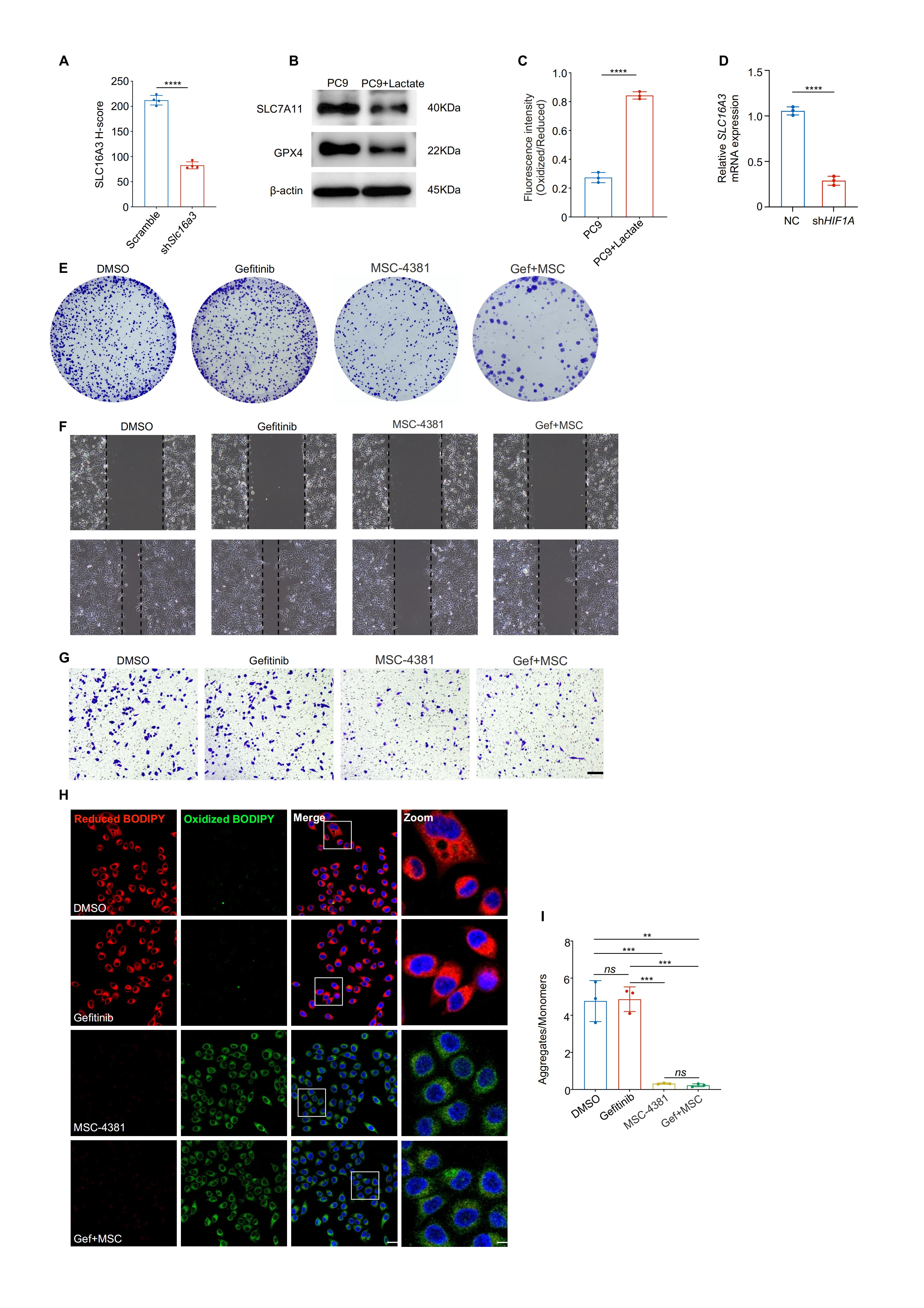

Supplement: Supplementary Figure 1 — Supporting data for ferroptosis regulation by SLC16A3 and therapeutic synergy of MSC-4381 with gefitinib. (A) Quantification of SLC16A3 immunohistochemical staining (H-score) in xenograft tumors from the scramble and shSLC16A3 groups (corresponding to Figure 2I). (B) Western blot analysis of ferroptosis-related proteins SLC7A11 and GPX4 in PC9 cells treated with exogenous lactate (10 mM) (corresponding to Figure 4H). (C) Quantification of lipid peroxidation (oxidized/reduced BODIPY C11 ratio) in lactate-treated PC9 cells (corresponding to Figure 4H). (D) qPCR analysis of SLC16A3 mRNA expression following HIF1A knockdown in PC9 cells (corresponding to Figure 5F). (E–G) Representative images of colony formation (E; corresponding to Figure 6F), wound healing (F; corresponding to Figure 6G), and Transwell invasion (G; corresponding to Figure 6H) assays in DMSO, gefitinib, MSC-4381, or gefitinib + MSC-4381 treatments. Scale bars, 100 μm. (H) Representative images of C11-BODIPY staining showing lipid ROS levels after the indicated treatments (corresponding to Figure 6H). Scale bars: 20 μm (left) and 5 μm (right). (I) Quantification of JC-1 red/green fluorescence ratio, indicating mitochondrial membrane potential changes (corresponding to Figure 6I). Data are presented as mean ± s.e.m.; ns, not significant; **P < 0.01, ***P < 0.001, ****P < 0.0001. [file Image1.jpg]

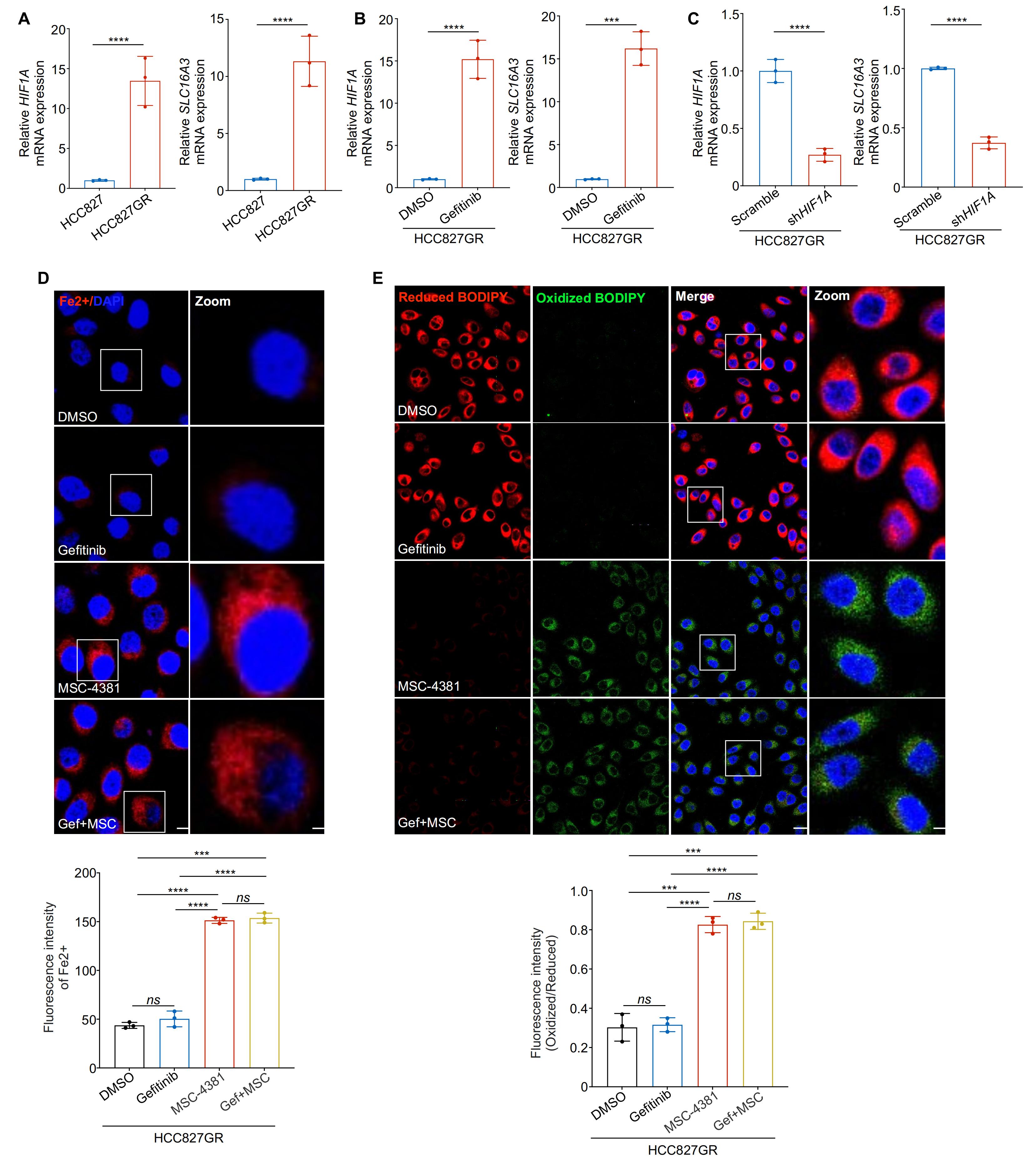

Supplement: Supplementary Figure 2 — Validation of the HIF1A-SLC16A3 axis and ferroptosis activation in an additional EGFR-mutant LUAD model (HCC827/HCC827GR). (A) qPCR analysis showing elevated HIF1A and SLC16A3 mRNA expression in HCC827GR compared with HCC827 cells (n = 3). (B) HIF1A and SLC16A3 mRNA levels were upregulated following gefitinib treatment in HCC827GR cells (n = 3). (C) Knockdown of HIF1A significantly decreased SLC16A3 expression in both HCC827 and HCC827GR cells (n = 3). (D) FerroOrange staining showing increased Fe2+ accumulation in HCC827GR cells upon gefitinib and MSC-4381 co-treatment (scale bar = 10 μm). Quantification of fluorescence intensity is shown below (n = 3). (E) C11-BODIPY 581/591 staining showing enhanced lipid peroxidation (oxidized/reduced ratio) after gefitinib and MSC-4381 co-treatment (scale bar = 10 μm). Quantification of fluorescence ratio is shown below (n = 3). Data are presented as mean ± SEM; ns, not significant; **P < 0.01, ***P < 0.001, ****P < 0.0001. [file Image2.jpeg]
