# Supplementary material for: SLC16A3 drives lung adenocarcinoma progression and gefitinib resistance through coordinated regulation of ferroptosis and lactate metabolism
Source: Front Immunol. 2025 Nov 10;16:1699540. doi: 10.3389/fimmu.2025.1699540 (PMC12640836; doi:10.3389/fimmu.2025.1699540)
Supplement: Supplementary file 3 [file Table1.docx]

**Supplementary Table S1 Primers sequences of RT-qPCR**

|  | F | R |
| --- | --- | --- |
| SLC16A3 | AGTGTGCGTGGTGTGGTCAG | CGAGGGCAGGTGGGTGTTAG |
| HIF1A | AGAGGTTGAGGGACGGAGAT | CTGTGCAGTGCAATACCTTCC |
| β-actin | CATGTACGTTGCTATCCAGGC | CTCCTTAATGTCACGCACGAT |

**Supplementary Table S2 Target sequence of shRNA and siRNA**

|  | F | R |
| --- | --- | --- |
| shSLC16A3#1 | GCGACTTGTTTATCCACTTTA | TAAAGTGGATAAACAAGTCGC |
| shSLC16A3#2 | CGTCTACATGTACGTGTTCAT | ATGAACACGTACATGTAGACG |
| shSLC16A3#3 | GCTCATACAGGAGTTTGGGAT | ATCCCAA ACTCCTGTATGAGC |
| shHIF1A | GTGATGAAAGAATTACCGAAT | ATTCGGTAATTCTTTCATCAC |
| siHIF1A | CGAUGGAAGCACUAGACAAAG | UUGUCUAGUGCUUCCAUCGGA |
| siKLF4 | GGACUUUAUUCUCUCCAAUTT | AUUGGAGAGAAUAAAGUCCTT |
| siMXI1 | GGAGAAGUGGACAAUAUAAGU | UUAUAUUGUCCACUUCUCCAU |
| siRORA | GGAAAGAGUUUAUGUUCUAUG | UAGAACAUAAACUCUUUCCAA |
| siZNF460 | GCUGAUGGUAUUUGUUCAAUG | UUGAACAAAUACCAUCAGCUG |
| siZNF682 | GGUGCUCGAGUCUUACUAAAC | UUAGUAAGACUCGAGCACCAG |

**Supplementary Table S3 Antibody information of western blotting**

| Antibody | Catalog Number | Supplier | Dilution |
| --- | --- | --- | --- |
| HIF-A | ab179483 | abcam | 1:1000 |
| SLC16A3 | AF5253 | Affinity | 1:500 |
| SLC7A11 | 32384-1-AP | Proteintech | 1:1000 |
| GPX4 | 67763-1-IG | Proteintech | 1:2000 |
| TFRC | AF5343 | Affinity | 1:500 |
| FSP1 | DF6516 | Affinity | 1:500 |
| DHODH | DF3991 | Affinity | 1:500 |
| β-actin | 200068-8F10 | Proteintech | 1:5000 |

**Supplementary Table S4 Antibody information of immunohistochemistry**

| Antibody | Catalog Number | Supplier | Dilution |
| --- | --- | --- | --- |
| SLC16A3 | AF5253 | Affinity | 1:200 |
| KI67 | 28074-1-AP | Proteintech | 1:80 |

**Supplementary Table S5 Antibody information of multiplex immunohistochemistry**

| Antibody | Catalog Number | Supplier | Dilution |
| --- | --- | --- | --- |
| SLC16A3 | AF5253 | Affinity | 1:200 |
| HIF-A | ab179483 | abcam | 1:200 |

**Supplementary Table S6. Oligonucleotide sequences used for dual-luciferase reporter assays**

| Construct | Sequence (5′→3′) | Description |
| --- | --- | --- |
| WT-HRE | TCACGT | Wild-type HIF1A binding site in the SLC16A3 promoter |
| Mut-HRE | AGTGCA | Mutated sequence disrupting HIF1A binding |
